# Risk of chronic kidney disease in patients with gout and the impact of urate lowering therapy: a population-based cohort study

**DOI:** 10.1186/s13075-018-1746-1

**Published:** 2018-10-30

**Authors:** Matthew Roughley, Alyshah Abdul Sultan, Lorna Clarson, Sara Muller, Rebecca Whittle, John Belcher, Christian D. Mallen, Edward Roddy

**Affiliations:** 1 East London NHS Foundation Trust, Trust Headquarters, 9 Alie Street, London, E1 8DE UK; 2 Research Institute for Primary Care and Health Sciences, Keele University, Keele, Staffordshire ST5 5BG UK; 3 School of Computing and Mathematics, Keele University, Keele, Staffordshire ST5 5BG UK; 4 Haywood Academic Rheumatology Centre, Midland Partnership NHS Foundation Trust, Haywood Hospital, Burslem, Staffordshire ST6 7AG UK

**Keywords:** Gout, Chronic kidney disease, Urate-lowering therapy, Cohort

## Abstract

**Background:**

An association between gout and renal disease is well-recognised but few studies have examined whether gout is a risk factor for subsequent chronic kidney disease (CKD). Additionally, the impact of urate-lowering therapy (ULT) on development of CKD in gout is unclear. The objective of this study was to quantify the risk of CKD stage ≥ 3 in people with gout and the impact of ULT.

**Methods:**

This was a retrospective cohort study using data from the Clinical Practice Research Datalink (CPRD). Patients with incident gout were identified from general practice medical records between 1998 and 2016 and randomly matched 1:1 to patients without a diagnosis of gout based on age, gender, available follow-up time and practice. Primary outcome was development of CKD stage ≥ 3 based on estimated glomerular filtration rate (eGFR) or recorded diagnosis. Absolute rates (ARs) and adjusted hazard ratios (HRs) were calculated using Cox regression models. Risk of developing CKD was assessed among those prescribed ULT within 1 and 3 years of gout diagnosis.

**Results:**

Patients with incident gout (*n* = 41,446) were matched to patients without gout. Development of CKD stage ≥ 3 was greater in the exposed group than in the unexposed group (AR 28.6 versus 15.8 per 10,000 person-years). Gout was associated with an increased risk of incident CKD (adjusted HR 1.78 95% CI 1.70 to 1.85). Those exposed to ULT had a greater risk of incident CKD, but following adjustment this was attenuated to non-significance in all analyses (except on 3-year analysis of women (adjusted HR 1.31 95% CI 1.09 to 1.59)).

**Conclusions:**

This study has demonstrated gout to be a risk factor for incident CKD stage ≥ 3. Further research examining the mechanisms by which gout may increase risk of CKD and whether optimal use of ULT can reduce the risk or progression of CKD in gout is suggested.

**Electronic supplementary material:**

The online version of this article (10.1186/s13075-018-1746-1) contains supplementary material, which is available to authorized users.

## Background

Gout is the most prevalent inflammatory arthritis, affecting 2.5% of adults in the UK and 3.9% in the USA [1, 2]. Chronic kidney disease (CKD) is also a common problem, with the global prevalence of CKD stages 3–5 (estimated glomerular filtration rate (eGFR) < 60 mL/min/1.73m^2^) estimated to be 10.6% [3]. An association between gout and CKD has been recognised for many years [4–6].

CKD can progress to end-stage renal disease (ESRD) and can lead to premature mortality [7]. The rate of progression to renal replacement therapy (RRT) or death over 5 years in patients with CKD stage 3 is 1.3% and 24.3%, respectively, and with stage 4 it is 19.9% and 45.7%, respectively [8]. In our recent systematic review and meta-analysis, 24% of people with gout had CKD stage ≥ 3 [9]. The association between hyperuricaemia, gout and CKD is thought to be bidirectional, with CKD known to be an independent risk factor for gout [10–13] and gout potentially predisposing to CKD by a number of mechanisms including hyperuricaemia, chronic inflammation and drug therapy with non-steroidal anti-inflammatory drugs (NSAIDs). In addition, hypertension, diabetes mellitus and obesity are highly prevalent in gout [14] and CKD, and are risk factors for CKD [15]. Our systematic review identified only two cohort studies investigating the risk of CKD in people with gout. Although large, both examined risk of ESRD rather than the earlier stages of CKD and neither used data from Europe [16, 17]. Better understanding of the risk of earlier stages of CKD in people with gout would help guide screening and the management of associated comorbidities and could aid the early identification or possible prevention of CKD in gout.

Urate-lowering therapy (ULT) should be considered for all patents with gout, in particular those with recurrent flares or tophi [18–20]. Data from randomised trials suggests that ULT in patients with CKD can slow the rate of decline of eGFR and reduce risk of progression to ESRD [21]. However, these trials were largely conducted in individuals without gout and the impact of ULT on development of CKD in people with gout remains unclear. The aim of this study was to quantify the risk of developing CKD stage ≥ 3 among patients with incident gout and assess the impact of ULT on this risk.

## Methods

### Data source and study population

This retrospective cohort study utilised data from the Clinical Practice Research Datalink (CPRD). The CPRD is a large database containing anonymised UK primary care medical records [22]. Approximately 98% of the population of England and Wales is registered with a general practitioner (GP), who is responsible for the majority of a patient’s medical care [23]. The CPRD covers more than 7% of the UK population and is representative of the general UK population in terms of age and gender distribution [23]. More than 58% of CPRD practices are linked to hospital episode statistics (HES). HES holds data items including admissions, diagnoses and operative procedures for all patients treated in hospitals in England [24]. The linkage is performed by a trusted third party based on National Health Service number, date of birth and gender. As HES only covers England; practices from Scotland, Wales and Northern Ireland were excluded from this analysis.

In this cohort study the exposed group consisted of individuals with a first-ever recorded diagnosis of gout and these were identified from general practice between 1998 and 2016 using previously published methods [25]. Ascertainment of gout was based on a medical (Read) code assigned by the GP. Gout diagnoses have been validated in the CPRD and have a positive predictive value of 90% [26]. Each patient with gout was assigned an index date corresponding to the date of gout diagnosis and randomly matched to one patient without a gout diagnosis or evidence of ULT, on age (± 5 years), gender, available follow-up time (± 3 years) and practice. Matching on follow up is a common approach when using the CPRD as patients with chronic illness typically have longer follow up compared to those without, and gout is associated with several comorbidities [25], it is a proxy method of minimising the potential bias this may induce. For both exposed and unexposed patients, follow up commenced from the index date. Those with evidence of CKD stage ≥ 3 or RRT before the index date or < 1 year after the index date were excluded from the study.

The primary outcome was developing CKD stage ≥ 3 and was based on two consecutive measurements of eGFR< 60 mL/min/1.73m^2^ at least 3 months apart. eGFR was calculated using serum creatinine values recorded in patients’ medical records using the Chronic Kidney Disease Epidemiology Collaboration equation [27]. For those considered to have CKD stage ≥ 3, the date of the first eGFR measurement was taken as the first occurrence of CKD. We also identified patients with CKD stage ≥ 3 or more based on a recorded diagnosis of CKD stages 3–5, ESRD or having evidence of renal replacement therapy (RRT (kidney transplant or dialysis)) in their primary or secondary care medical record.

### Covariates

To assess the independent association between gout and CKD stage ≥ 3, information on various baseline characteristics was extracted. These included body mass index (BMI), smoking status, index of multiple deprivation (IMD), and specific comorbidities. The comorbidities included were; myocardial infarction, systemic lupus erythematosus (SLE), rheumatoid arthritis, congestive heart failure, cerebrovascular disease, peripheral vascular disease, hospitalisations and treated hypertension or diabetes mellitus before the index date. Information was extracted on NSAID use (two or more prescriptions) in the 6 months before gout diagnosis. In addition, baseline serum uric acid (SUA) level was adjusted for in the analyses examining risk of CKD associated with ULT prescription. Finally, for each subject we calculated the visit rate on unique calendar dates with a medical diagnosis code over the observation time to estimate how often they visited their general practitioner. The visit rate was then categorised into tertiles.

**Fig. 1:**
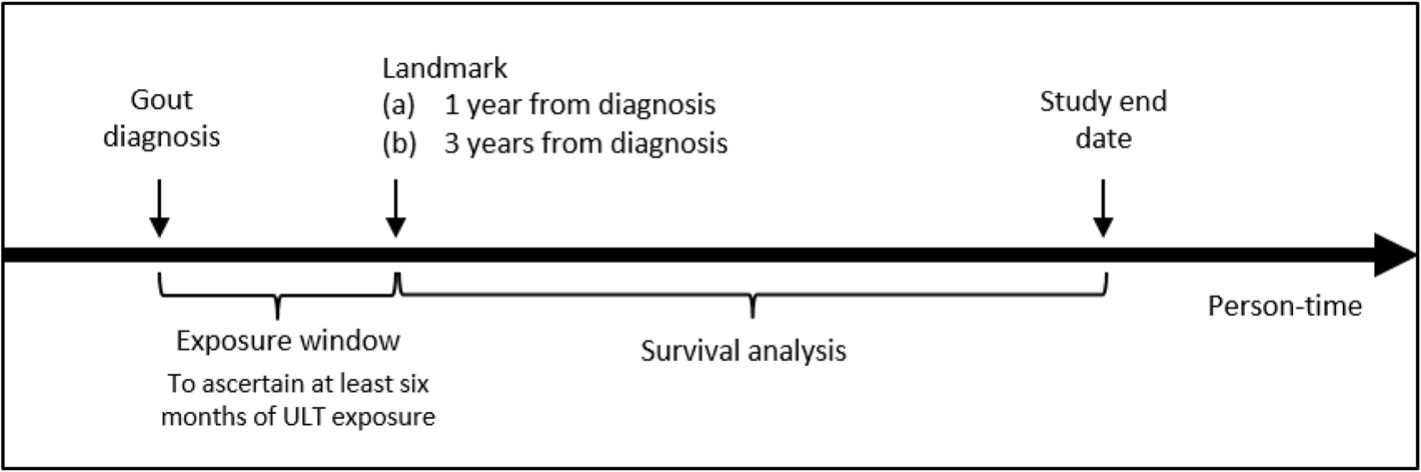
Graphical illustration of landmark analysis. ULT, urate-lowering therapy

Landmark analysis is routinely used to assess the impact of treatment where there is a potential lag between disease occurrence and initiation of therapy [28]. As the timing of initiation of ULT varies after gout diagnosis, we utilised landmark analysis to examine the effect of ULT on the risk of CKD. Landmark analysis deals with the issue of immortal time bias, which biases the results in favour of the treatment under study by granting a spurious survival advantage to the treated group [28]. In the case of gout, patients receiving ULT must have at least survived from time of diagnosis to time of treatment whereas no such requirement is necessary for the unexposed group (individuals with gout not receiving ULT). Bias would be introduced by ignoring this, as ULT exposure status may be dependent on the length of follow up. In landmark analysis, a fixed time after the initiation of therapy is selected a priori for conducting survival analysis [29]. Only those alive, event-free and contributing data at the landmark time were included in the analysis. Exposure to ULT was evaluated between the index date (diagnosis of gout) and the landmark time, whereas development of CKD stage ≥ 3 was only considered after the landmark time point. Two landmark points were considered in the analysis (1 and 3 years after diagnosis) based on a previously published study [30]. Only patients initiated on and prescribed more than 6 months of ULT were considered to be exposed (Fig. 1). This was based on previous literature [30] and expert consensus, as allopurinol is started at a low dose and increased gradually and it can take several months to escalate the dose sufficiently to lower serum urate to below the biochemical target level. The duration of ULT was calculated based on quantity prescribed and numeric daily dose.

### Statistical analysis

Absolute rates (ARs) of CKD stage ≥ 3 per 10,000 person-years and 95% confidence intervals (CI) were calculated for the exposed and unexposed groups. These were stratified by age, gender, IMD and time after diagnosis. Hazard ratios (HRs) were modelled using Cox proportional hazards regression adjusting for the stated confounding factors. Those with missing body mass index (BMI) status were categorised separately and included in the analysis, as BMI was assumed not to be missing at random. Similarly, we compared the risk of CKD stage ≥ 3 among those prescribed ULT within 1 and 3 years after diagnosis to patients with gout who were not prescribed ULT. The HRs were additionally adjusted for baseline serum creatinine and uric acid levels. Baseline serum creatinine and uric acid level was considered before the ULT exposure or landmark date for those not prescribed ULT. For those with missing laboratory values, an indicator variable was included in the regression analysis. All missing values were imputed using a constant to ensure that all data were included in the analysis. This study was approved by the CPRD in-house Independent Scientific Advisory Committee (ISAC) reference number 15_214RA.

Sample size calculations: based on previous literature, we anticipated at least 30,000 cases of incident gout in HES-linked CPRD matched to a similar number of unexposed individuals [31]. Given the annual incidence of stage 3 CKD is 15% (aged 65–74 years) in the UK, our sample size provided more than 99% power to detect a HR of 1.5 using Cox proportional hazards model at 5% level of significance. For the landmark analysis, assuming that 10% of patients with gout are treated with ULT within the first year, we had approximately 82% power to detect a HR of 1.35 between ULT users and non-users, using a Cox proportional hazards model at 5% level of significance.

## Results

Patients with incident gout (*n* = 41,446) were identified and matched to 41,446 patients without gout. At baseline, mean participant age was 57 years and 81% were male. The median duration of follow up was 6 years with a total of 484,455 person-years of follow up. At baseline, patients with gout had a higher prevalence of diabetes mellitus, hypertension, vascular disease and obesity. In addition, patients with gout attended their GP more frequently and received more NSAID prescriptions than patients without gout (Table 1).

**Table 1:** Basic characteristics of the study population

Variable	Gout		Non-gout	
	Number	Percentage	Number	Percentage
Total number	41,446		41,446	
Mean age (SD)	57.2	(13.6)	57.1	(13.7)
Median follow up (IQR)	6.0	(3.3, 9.5)	5.9	(3.2, 9.4)
Male	33,574	81.0	33,574	81.0
Body mass index				
Normal	7394	17.8	12,341	29.8
Underweight	349	0.8	681	1.6
Overweight	15,537	37.5	14,760	35.6
Obese	15,311	36.9	8417	20.3
Missing	2855	6.9	5247	12.7
Smoking status				
Never/ex-smoker	36,153	87.2	34,406	83.0
Current smoker	5293	12.8	7040	17.0
Comorbidities				
Diabetes mellitus	2686	6.5	2417	5.8
Treated hypertension	11,982	28.9	6648	16.0
Rheumatoid arthritis	276	0.7	302	0.7
SLE	25	0.1	24	0.1
Heart failure	1342	3.2	482	1.2
Myocardial infarction	1660	4.0	1166	2.8
Cerebrovascular disease	1537	3.7	1241	3.0
Peripheral vascular disease	901	2.2	670	1.6
Anti-diabetic drugs	1847	4.5	1881	4.5
NSAIDs	5852	14.1	1619	3.9
Previous hospitalisations	11,016	26.6	9129	22.0
GP consultation rates (tertiles)				
1	10,375	25.0	17,256	41.6
2	14,609	35.2	13,022	31.4
3	16,462	39.7	11,168	26.9
IMD quintiles				
1 (least deprived)	10,526	25.4	10,485	25.3
2	10,220	24.7	10,232	24.7
3	8411	20.3	8330	20.1
4	7034	17.0	7164	17.3
5 (most deprived)	5216	12.6	5206	12.6

*SLE* systemic lupus erythematosus, *NSAID* non-steroidal anti-inflammatory drug, *GP* general practitioner, *IMD* Index of multiple deprivation

During follow up, 6694 patients (16.2%) with gout developed CKD stage ≥ 3 compared to 3953 (9.5%) patients without gout (absolute rate 28.6 versus 15.8 per 10,000 person-years respectively). A diagnosis of gout was associated with increased risk of development of CKD stage ≥3 compared to patients without gout (unadjusted HR 1.79 95% CI 1.72 to 1.86). Adjustment for age, gender, comorbidities, deprivation, NSAID use, frequency of hospital admission and GP attendance, had a minimal effect and the association remained statistically significant (adjusted HR 1.78 95% CI 1.70 to 1.85) (Table 2).

**Table 2:** Absolute rate of CKD per 10,000 person-years and hazard ratios

Variable	Gout			Non-gout			Unadjusted		Adjusted*	
	*n*	Rate^‡^	95% CI	*n*	Rate^‡^	95% CI	Hazard ratio	95% CI	Hazard ratio	95% CI
Overall	6694	28.6	27.9, 29.3	3953	15.8	15.3, 16.3	1.79	1.72, 1.86	1.78	1.70, 1.85
Male	4608	23.6	22.9, 24.3	2681	13.0	12.5, 13.5	1.80	1.71, 1.89	1.78	1.69, 1.87
Female	2086	53.8	51.5, 56.1	1272	28.7	27.1, 30.3	1.82	1.70, 1.95	1.79	1.66, 1.93
Age at index in years										
< 55 years	690	5.8	5.4, 6.30	279	2.3	2.1, 2.6	2.52	2.19, 2.89	1.78	1.54, 2.07
55–65	1581	24.6	23.4, 25.8	844	12.3	11.5, 13.2	1.99	1.83, 2.16	1.76	1.61, 1.92
65–75	2506	66.2	63.6, 68.8	1498	33.8	32.1, 35.5	1.91	1.79, 2.04	1.87	1.75, 2.00
> 75	1917	141.0	134.8, 147.5	1332	78.1	74.0, 82.4	1.75	1.63, 1.88	1.71	1.59, 1.84
IMD (quintiles)										
1 (least deprived)	1579	25.6	24.3, 26.9	914	13.9	13.0, 14.8	1.81	1.67, 1.97	1.84	1.69, 2.01
2	1689	29.2	27.9, 30.7	1008	16.2	15.2, 17.2	1.78	1.65, 1.92	1.79	1.65, 1.94
3	1439	30.9	29.3, 32.5	831	16.7	15.6, 17.9	1.83	1.68, 1.99	1.77	1.62, 1.94
4	1143	29.4	27.8, 31.2	698	16.6	15.4, 17.8	1.76	1.60, 1.93	1.78	1.61, 1.97
5 (most deprived)	841	29.1	27.2, 31.2	501	16.5	15.1, 18.0	1.76	1.57, 1.96	1.67	1.49, 1.88

*CKD* chronic kidney disease, *IMD* index of multiple deprivation

*Adjusted for age, gender, body mass index, smoking status, diabetes mellitus, treated hypertension, rheumatoid arthritis, systemic lupus erythematosus, heart failure, IMD, myocardial infraction, cerebrovascular disease, peripheral vascular disease, history of hospitalisation, consultation rates, and non-steroidal anti-inflammatory drug exposure, when not stratified by them, ^‡^ per 10,000 person-years

**Fig. 2:**
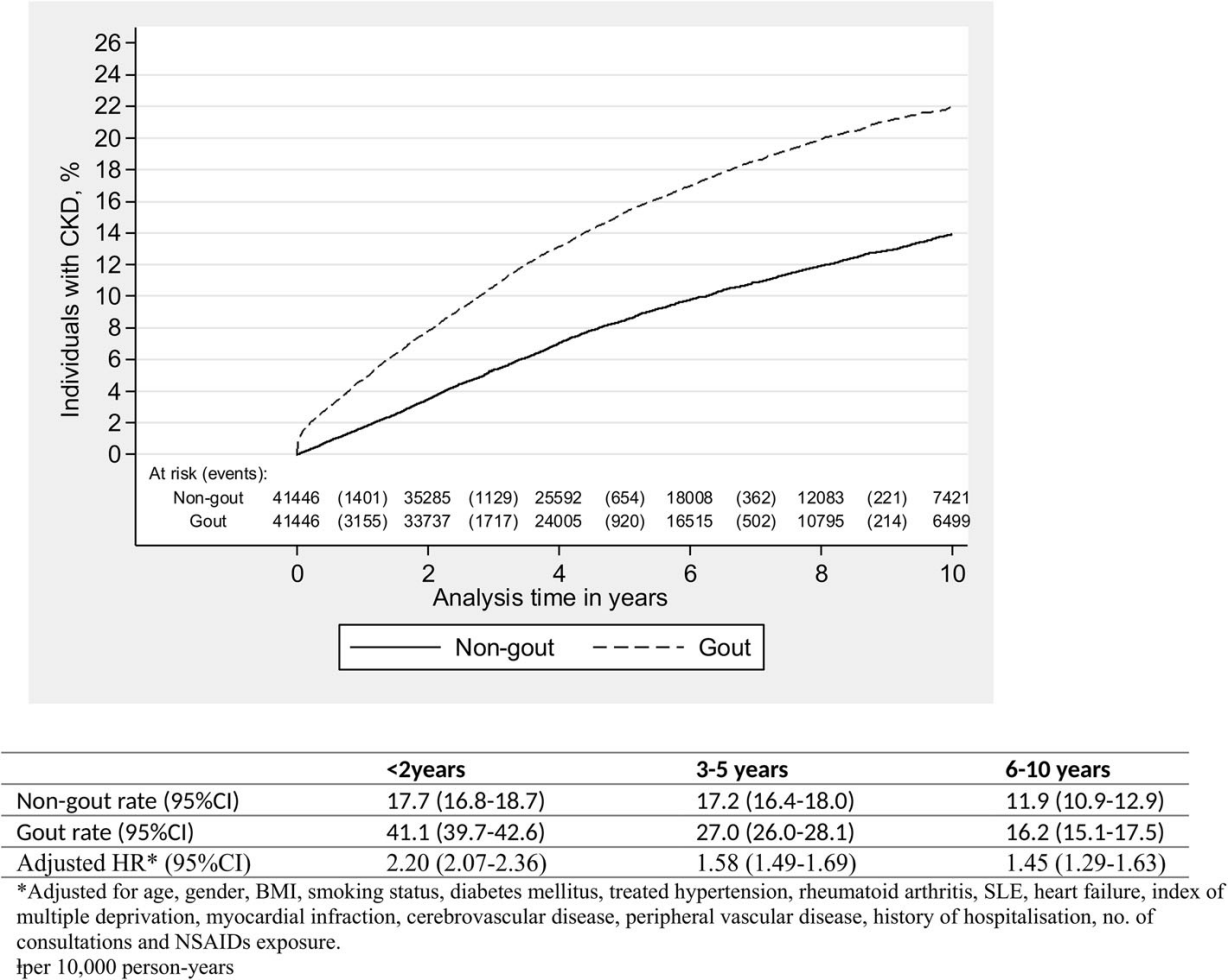
Development of chronic kidney disease (CKD) stage ≥ 3 in patients with gout and patients without gout (non-gout) during follow up

In the stratified analyses, for both exposed and unexposed patients, the absolute rate of development of CKD stage ≥ 3 was greater in women and increased with age. The adjusted HRs remained largely consistent between genders and across all age groups and IMD quintiles (Table 2). Risk of development of CKD stage ≥ 3 was found to be higher within the first 2 years of gout diagnosis (adjusted HR 2.20 95% CI 2.07 to 2.36) compared to 6–10 years following diagnosis (adjusted HR 1.45 95% CI 1.29 to 1.63). Figure 2 describes the development of CKD stage ≥ 3 in patients with gout and patients without gout during follow up.

In the landmark analysis, patients with gout were excluded due to either death, developing CKD or transfer from general practice within 1 year (*n* = 1962) or 3 years (*n* = 12,947) of gout diagnosis. Of the remaining patients with gout, 4198 (10.6%) in the 1-year landmark analysis and 4793 (16.8%) in the 3-year landmark analysis were receiving ULT (Additional file 1: Figure S1).

Those receiving ULT were older, more frequently hypertensive and diabetic and had higher baseline serum urate levels compared to those unexposed to ULT (Table 3). Those exposed to at least 6 months of ULT within 1 and 3 years of gout diagnosis had a greater risk of development of CKD stage ≥ 3, compared to those not exposed (1-year unadjusted HR 1.47 95% CI 1.35 to 1.59, 3-year unadjusted HR 1.35 95% CI 1.23 to 1.49). This risk however, following adjustment, was attenuated to non-significance in all analyses apart from the 3-year landmark analysis in women only (adjusted HR 1.31 95% CI 1.09 to 1.59) (Table 4).

**Table 3:** Basic characteristics of gout cases by ULT exposure with 1 and 3 years after gout diagnosis

Variable	1-Year landmark				3-Year landmark			
	Exposed *n* = 4198		Unexposed *n* = 35,286		Exposed *n* = 4793		Unexposed *n* = 23,706	
	*N*	%	*N*	%	*N*	%	*N*	%
Mean age (SD)	58.2	(12.8)	56.3	(13.5)	56.1	(12.1)	55.1	(12.9)
Male	3485	83.0	28,809	81.6	4151	86.6	19,526	82.4
Body mass index								
Normal	530	12.6	6385	18.1	575	12.0	4335	18.3
Underweight	22	0.5	292	0.8	15	0.3	196	0.8
Overweight	1504	35.8	13,294	37.7	1781	37.2	9036	38.1
Obese	1893	45.1	12,801	36.3	2177	45.4	8581	36.2
Missing	249	5.9	2514	7.1	245	5.1	1558	6.6
Smoking status								
Never/ex-smoker	3808	90.7	30,501	86.4	4355	90.9	20,459	86.3
Current smoker	390	9.3	4785	13.6	438	9.1	3247	13.7
Comorbidities								
Diabetes mellitus	357	8.5	2114	6.0	338	7.1	1259	5.3
Treated hypertension	1539	36.7	9509	26.9	1625	33.9	5856	24.7
Rheumatoid arthritis	40	1.0	213	0.6	39	0.8	122	0.5
Heart failure	200	4.8	900	2.6	166	3.5	372	1.6
Myocardial infraction	219	5.2	1247	3.5	216	4.5	685	2.9
Cerebrovascular disease	176	4.2	1172	3.3	143	3.0	627	2.6
Peripheral vascular disease	118	2.8	663	1.9	85	1.8	351	1.5
Anti-diabetic drugs	233	5.6	1462	4.1	208	4.3	860	3.6
NSAIDs	1082	25.8	4455	12.6	1143	23.8	2887	12.2
Previous hospitalisations	1256	29.9	8964	25.4	1200	25.0	5392	22.7
IMD quintiles								
1	871	20.7	9211	26.1	1089	22.7	6338	26.7
2	1021	24.3	8700	24.7	1178	24.6	5894	24.9
3	889	21.2	7076	20.1	977	20.4	4733	20.0
4	764	18.2	5935	16.8	827	17.3	3915	16.5
5	647	15.4	4332	12.3	717	15.0	2810	11.9
Mean serum urate (SD) μmol/L	478.4	(90.8)	432.2	(98.1)	476.5	(95.7)	427.3	(99.1)
Mean serum creatinine (SD)* μmol/L	89.5	(16.7)	87.2	(16.1)	90.3	(15.2)	86.4	(15.4)

*NSAID* non-steroidal anti-inflammatory drug, *IMD* Index of multiple deprivation

*Missing serum creatinine value = 10,335 (1-year landmark), 5872 (3-year landmark), missing serum urate: 15,638 (1-year landmark), 10,176 (3-year landmark)

**Table 4:** Absolute rate of CKD by ULT exposure

Variable	Exposed			Unexposed			Unadjusted		Adjusted*	
	*n*	Rate^‡^	95% CI	*n*	Rate^‡^	95% CI	Hazard ratio	95% CI	Hazard ratio	95% CI
1-Year landmark										
Overall	674	34.3	31.8, 37.0	4058	23.3	22.6, 24.0	1.47	1.35, 1.59	1.09	0.99, 1.18
Male	450	26.4	24.1, 28.9	2878	19.8	19.1, 20.5	1.33	1.21, 1.47	1.08	0.98, 1.20
Female	224	86.7	76.1, 98.9	1180	41.0	38.7, 43.4	2.01	1.74, 2.32	1.11	0.96, 1.29
3-Year landmark										
Overall	549	26.1	24.0, 28.4	2027	19.3	18.4, 20.1	1.35	1.23, 1.49	1.03	0.94, 1.14
Male	390	20.7	18.8, 22.9	1538	17.5	16.6, 18.4	1.19	1.06, 1.33	0.96	0.85, 1.07
Female	159	71.1	60.9, 83.1	489	28.3	25.9, 31.0	2.43	2.03, 2.90	1.31	1.09, 1.59

*CKD* chronic kidney disease, *ULT* urate-lowering therapy

*Adjusted for age, gender, body mass index, smoking status, diabetes mellitus, treated hypertension, rheumatoid arthritis, heart failure, index of multiple deprivation, myocardial infarction, cerebrovascular disease, peripheral vascular disease, history of hospitalisation, non-steroidal anti-inflammatory drug exposure and baseline serum creatine and uric acid, when not stratified by them. ^‡^ per 10,000 person-years

## Discussion

This retrospective cohort study, set in a large UK primary care population, compared the risk of developing CKD stage ≥ 3 in those with gout versus those without gout. Following adjustment for age, gender, comorbidities, deprivation, NSAID use, frequency of hospital admission and GP attendance, patients with gout had 78% increased risk of development of CKD stage ≥ 3 compared to patients without gout. Risk of CKD development was highest in the first 2 years following gout diagnosis. Following adjustment patients with gout exposed to at least 6 months ULT had no increased risk of developing CKD compared to those not exposed, in all analyses apart from analysis in women receiving ULT within 3 years of diagnosis.

This study has a number of strengths. Participants were from primary care where the majority of patients with gout are managed, thus aiding generalisability. The sample size was large and the median follow up was 6 years, which should be sufficient for development and ascertainment of CKD stage ≥ 3. Ascertainment of the primary outcome required either a clinical diagnostic code or two consecutive eGFR measurements < 60 mL/min/1.73m^2^. Utilising biochemical data and Read codes should aid completeness compared to using codes alone, as GP coding of CKD has been shown to capture only 72% of those with biochemically evident disease [32]. Previous cohort studies examining gout and renal disease used either record linkage or diagnostic codes alone and examined either the severest form of CKD (ESRD) [16, 17] or “renal diseases” [25], which would include a large number of heterogenous conditions. This is the first study to the best of our knowledge to examine risk of earlier stages of CKD and to use biochemical data, which is an additional strength. Immortal time bias, which could have resulted in lower observed risk of CKD associated with ULT exposure, was addressed with the use of landmark analysis, which is also a strength of this study.

An important caveat is gout ascertainment based on GP-coded diagnoses alone, risking misclassification bias, although gout diagnoses have been validated in CPRD and have a positive predictive value of 90% [26]. Ascertainment bias is a possible limitation of this study as patients with gout presented more frequently to their GP and hospital and had higher prevalence of hypertension and diabetes mellitus, which could have prompted more frequent renal function testing. GP consultation rates during follow up were adjusted for in the statistical analysis but may not completely address this issue. Furthermore, it was not possible to account for patient ethnicity or the severity of comorbidities. Regarding ULT prescription data, prescriptions do not necessarily equate to dispensing of ULT and it was not possible assess adherence.

In this study, those with CKD stage ≥ 3 or RRT occurring pre-index or within 1 year of gout diagnosis were excluded. Despite this, the possibility of reverse causation could still potentially underlie an association between gout and CKD e.g. undiagnosed or mild renal dysfunction leading to hyperuricaemia, thus conferring risk of gout development, with later progression to CKD [33]. It is possible that our finding of the risk of CKD development being highest within 2 years of gout diagnosis reflects this. It is also of note that nine genetic loci associated with both CKD and serum urate concentration, with varying direction of effect, have been identified by genome-wide association studies, which could further complicate the relationship between gout and CKD [34].

The prevalence of CKD stage ≥ 3 in gout was found to be 24% in our recent systematic review and meta-analysis [9]. We identified only two other prospective studies examining the risk of CKD associated with gout. These studies reported an increased risk of ESRD of 57% [17] and 80% [16], in keeping with our risk estimate for CKD stage ≥ 3. One study published subsequent to our systematic review found three times increased risk of “renal diseases” (defined using Read codes rather than eGFR) following gout diagnosis but did not differentiate between acute or chronic forms [25]. In our study allopurinol accounted for 99% of all ULT prescriptions. We did not find clear evidence that ULT exposure influenced the risk of developing CKD. Risk was greater in those exposed to ULT, but those exposed were older and more frequently had diabetes mellitus and hypertension and these factors appeared to explain the ULT-CKD association in our data. In previous studies examining the association between ULT and renal disease, benefits were noted to be greatest in those taking higher doses of ULT [35] or reaching target SUA levels [36]. It is of note, however, that patients with gout often remain on lower doses of allopurinol and the majority do not reach target SUA levels [37, 38]. This study has not explored whether target SUA levels were reached and our finding of no association may reflect suboptimal urate-lowering rather than the true effect of ULT.

Women who develop gout are typically older, have more comorbidities such as hypertension, diabetes mellitus and obesity and receive ULT less frequently than men [39]. Possible explanations for our finding of increased risk of CKD associated with ULT in women in the 3-year analysis include women prescribed ULT potentially having more severe gout and therefore possibly conferring greater risk of CKD, incomplete adjustment for comorbidities or medications or ascertainment bias, as comorbid women taking allopurinol may have more frequent renal function testing. It is possible that allopurinol has deleterious effects on renal function in women with gout but to the best of our knowledge this has not been found in previous studies. The finding of increased risk was not replicated in the 1-year analysis, however, suggesting the finding in the 3-year analysis could be related to chance.

Whilst it is not possible to make causal inferences from this observational study, it is worth considering the potentially plausible mechanisms for the association between gout and CKD. Renal damage could result from comorbid hypertension, diabetes mellitus, obesity or use of nonsteroidal anti-inflammatory drugs. Hyperuricaemia-mediated endothelial dysfunction has been suggested to lead to renovascular disease [40], although Mendelian randomisation studies have not found an association between urate and CKD [34]. Inflammation in gout is increasingly recognised to persist in the intercritical period between acute attacks [41, 42], raising the possibility that inflammatory mechanisms contribute to increased risk. Activation of the NLRP3 inflammasome and subsequent production of interleukin-1β is a key inflammatory process in gout [43]. This is of note as renal NLRP3 expression is significantly increased in CKD and it has been suggested that this and interleukin-1β contribute to progression of CKD [44, 45]. We are unable to make comparisons to previous cohort studies, as they have used different outcome measures and, as discussed above, the possibility of reverse causation complicates temporal inferences from this study. As also noted previously, a number of conditions associated with gout are also risk factors for CKD and incomplete adjustment for these could result in residual confounding.

## Conclusion

This study has demonstrated gout to be a risk factor for incident CKD stage ≥ 3, after adjustment for age, gender, comorbidities, deprivation, NSAID use, frequency of hospital admission and GP attendance. In clinical practice, renal function monitoring is often suboptimal in gout [36] suggesting an area for improvement. Further research examining the mechanisms by which gout may increase risk of CKD is suggested, including the role of hyperuricaemia and possible linked inflammatory processes. Due to high prevalence of CKD in gout, further research into whether optimal use of ULT can reduce the risk or progression of CKD in patients with gout would also be of value.

## Additional file

Additional file 1: **Figure S1.** Landmark analysis. (A) 1-year landmark. (B) 3-year landmark. (TIF 1482 kb)

## Abbreviations

AR: Absolute rate
BMI: Body mass index
CKD: Chronic kidney disease
CPRD: Clinical practice research datalink
eGFR: Estimated glomerular filtration rate
ESRD: End-stage renal disease
GP: General practitioner
HES: Hospital episode statistics
HR: Hazard ratio
IMD: Index of multiple deprivation
ISAC: Independent scientific advisory committee
NSAIDs: Non-steroidal anti-inflammatory drugs
RRT: Renal replacement therapy
SLE: Systemic lupus erythematosus
SUA: Serum uric acid
ULT: Urate-lowering therapy
