# Supplementary figures and images for: Risk of chronic kidney disease in patients with gout and the impact of urate lowering therapy: a population-based cohort study
Source: Arthritis Res Ther. 2018 Oct 30;20:243. doi: 10.1186/s13075-018-1746-1 (PMC6235219; doi:10.1186/s13075-018-1746-1)

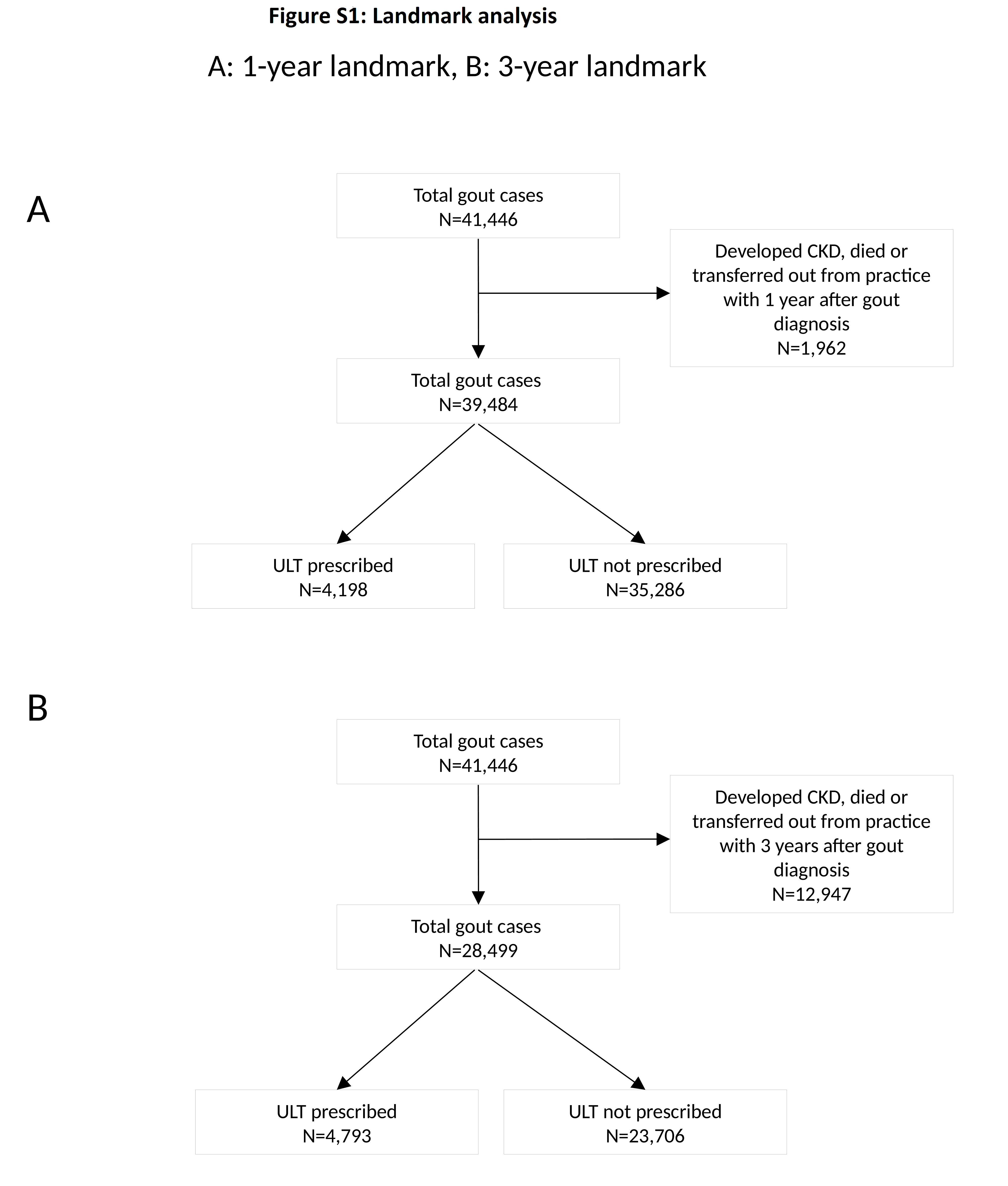

Supplement: Supplementary file 1 — Figure S1. Landmark analysis. (A) 1-year landmark. (B) 3-year landmark. (TIF 1482 kb) [file 13075_2018_1746_MOESM1_ESM.tif]
